# Characterization and Interaction with Biomembrane Model of Benzo[k,l]xanthene Lignan Loaded Solid Lipid Nanoparticles

**DOI:** 10.3390/membranes12060615

**Published:** 2022-06-13

**Authors:** Cristina Torrisi, Nunzio Cardullo, Vera Muccilli, Corrado Tringali, Francesco Castelli, Maria Grazia Sarpietro

**Affiliations:** 1 Department of Drug and Health Sciences, University of Catania, Viale Andrea Doria 6, 95125 Catania, Italy; torrisi.cristina@hotmail.it (C.T.); fcastelli@unict.it (F.C.); 2 Department of Chemical Sciences, University of Catania, Viale Andrea Doria 6, 95125 Catania, Italy; ncardullo@unict.it (N.C.); v.muccilli@unict.it (V.M.); ctringali@unict.it (C.T.)

**Keywords:** SLN, benzo[k,l]xanthene lignans, DSC, MLV, biomembrane model

## Abstract

Benzo[k,l]xanthene lignans are a group of rare natural products belonging to the class of polyphenols with promising biological activities and are studied as potential chemotherapeutic agents. The lipophilic character of a xanthene core makes these molecules difficult to be used in an aqueous medium, limiting their employment in studies for pharmaceutical applications. To overcome this problem, a drug-delivery system which is able to improve the stability and bioavailability of the compound can be used. In this study, a bioactive benzoxanthene lignan (BXL) has been included in SLN. Unloaded and BXL-loaded SLN have been prepared using the Phase Inversion Temperature method and characterized in terms of size, zeta potential, entrapment efficiency and stability. Differential scanning calorimetry was used to evaluate the thermotropic behavior and ability of SLN to act as carriers for BXL. A biomembrane model, represented by multilamellar vesicles, was used to simulate the interaction of the SLN with the cellular membrane. Unloaded and loaded SLN were incubated with the MLV, and their interactions were evaluated through variations in their calorimetric curves. The results obtained suggest that SLN could be used as a delivery system for BXL.

## 1. Introduction

Benzo[k,l]xanthene lignans (BXLs) are a group of rare natural products belonging to the class of polyphenols. To date, only six naturally occurring BXLs have been discovered: rufescidride [1], mongolicumin A [2] and its dimethyl ester [3], yunnaneic acid H [4], chiliantin D [5], and dodegranoside [6]. Because of their limited availability in nature, the biological activities of natural BXLs have been almost unexplored for years. In 2009, a simple and biomimetic methodology for the synthesis of BXLs was published [7], and a variety of synthetic benzoxanthenes have since been obtained and evaluated as antioxidant [8], anti-inflammatory [9], selective copper-chelators [10], antifungal [11], antibacterial [12], antiproliferative agents [13,14]. Additionally, BXLs are reported to induce autophagy towards tumor cells [15] and act as antiangiogenic [16], pro-apoptotic agents [17], DNA-binders [12,17], proteasome inhibitors [18], all targets involved in tumorigenesis, and which are useful for the development of anticancer drugs. These studies highlighted the promising biological activities of this class of lignans as potential chemotherapeutic agents. Nevertheless, the lipophilic character of the xanthene core [14] makes these molecules difficult to use in an aqueous medium, limiting their employment in in vivo studies for pharmaceutical applications. One of the approaches to overcome this problem is the employment of a drug-delivery system able to improve the stability and bioavailability of the candidate drug. In recent years, solid lipid nanoparticles (SLN) have attracted the attention of numerous researchers as carriers for lipophilic molecules such as BXLs. SLN are colloidal systems composed of solid lipids at room and body temperature and are stabilized by surfactants. They represent an alternative option to the traditional colloidal drug-delivery systems, such as emulsions, liposomes, polymeric micro- and nanoparticles, because of the reduced problems related to industrial production scale-up and mid-term storage. Some of their main advantages are related to their nanometric size, between 50 and 1000 nm, which allows them to be used for different routes of administration and in the presence of biocompatible lipids that make them safe and efficient delivery vehicles. As described in the literature, SLN can be produced by different methods and using various lipid compositions that strongly affect their drug-loading capacity and drug release profile [19,20]. In this preliminary study, a bioactive benzoxanthene (BXL) has been included in SLN. Unloaded and BXL-loaded SLN have been prepared using the Phase Inversion Temperature (PIT) method and characterized in terms of size, zeta potential, entrapment efficiency and stability. Their thermotropic behavior and interaction with biomembrane models, made of dimyristoylphosphatidylcholine multilamellar vesicles (MLV), was evaluated using differential scanning calorimetry. The release profile of bioactive compound from BXL-loaded SLN was evaluated with an in vitro model. Finally, two spectroscopic assays were performed to evaluate the effect of drug encapsulation on BXL’s antioxidant activity.

## 2. Materials and Methods

### 2.1. Materials

Precirol^®^ ATO 5 (Glyceryl distearate) was kindly donated by Gattefossé (Saint-Pries, France). TEGIN^®^ O (Glyceriloleate) and Oleth-20 were obtained from A.C.E.F. S.p.a (Piacenza, Italy). Dimyristoylphospatidylcholine (DMPC) was obtained from Genzyme (Liestal, Switzerland). Caffeic acid and Mn(OAc)_3_* 2 H_2_O were purchased from Sigma Aldrich (Milan, Italy). Purified water from Millipore-Q^®^ Gradient A10TMultra-pure watersystem (Millipore, Guyancourt, France) was used throughout the study.

### 2.2. Synthesis of BXL

**Scheme 1:**
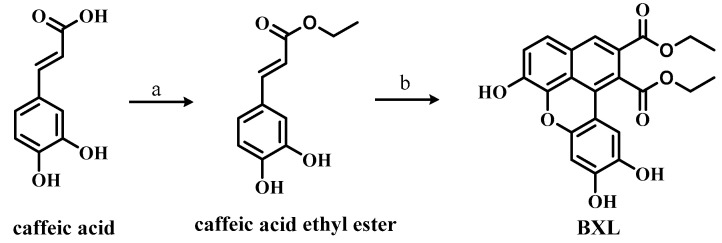
Synthesis of BXL (a) H_2_SO_4_, ethanol, reflux, 12 h; (b) Mn(OAc)_3_, CHCl_3_, rt, 3 h.

The benzoxanthene lignan diethyl 6,9,10-trihydroxybenzo[k,l]xanthene-1,2-dicarboxylate (to the follow simply BXL) was synthesized according to the methodology previously reported [14]. Briefly, caffeic acid (400 mg, 2.2 mmol) was refluxed with an excess of EtOH (70 mL) and a catalytic amount of concentrated H_2_SO_4_ (0.2 mL) for 24 h. The mixture was concentrated, diluted with 50 mL of EtOAc and partitioned with a saturated NaHCO_3_ solution (50 mL). The aqueous phase was partitioned with EtOAc (3 × 50 mL), and the combined organic layers were dried over anhydrous Na_2_SO_4_, filtered and taken until dry. The caffeic acid ethyl ester was recovered from the organic phase with 92% yield without further purification. The ester (475 mg, 2.0 mmol) was solubilized in CHCl_3_ (50 mL) and a suspension of Mn(OAc)_3_ (2.170 gr, 8.0 mmol; 50 mL of CHCl_3_) was added. The mixture was stirred for 12 h and then a saturated ascorbic solution (40 mL) was added to quench the reaction. The two phases were partitioned, and the aqueous layer was partitioned again with CH_2_Cl_2_ (3 × 40 mL). The recovered organic layer was subjected to column chromatography on diol silica gel eluting with CH_2_Cl_2_:MeOH (100:0 → 95:5), thus affording BXL with 58% yield (Scheme 1). NMR data were in perfect agreement with those previously reported [14].

### 2.3. Preparation of SLN

Unloaded and BXL-loaded SLN, whose composition is reported in Table 1, were prepared using the PIT method. Briefly, the lipid phase containing Precirol ATO 5, the emulsifiers (Oleth-20 and Tegin O) and the aqueous phase were separately heated on a magneto–thermal plate at 75–80 °C. When the two phases reached the same temperature, the aqueous phase was added drop by drop, at constant temperature and under agitation, to the oil phase. The opaque formulation became transparent, realizing the phase reversal from an A/O system to an O/A system. After that, the formulation was taken to room temperature under stirring. BXL was added to the oil phase.

### 2.4. SLN Physicochemical Characterization

The average size (Z-Ave) and polydispersity index (PDI) of SLN were measured by Dynamic Lights Scattering (DLS) method, using a Zeta Sizer Nano-ZS90 (Malvern Instrument Ltd., Worcs, England). The instrument was equipped with a laser whose nominal power was 4.5 mW with a maximum power of 5 mW at 670 nm. The analysis was performed using a 90° scattering angle at 25 + 0.2 °C. Before the measurements, 40 µL of each sample suspension was diluted in1 mL of deionized water. Measurements were performed in triplicate and the calculated mean values were used. The zeta potential (ZP, ξ) was measured by Electrophoretic Light Scattering (ELS) using a Zeta Sizer Nano-ZS90 (Malvern Instrument Ltd., Worcs, England). Each measurement was recorded at 25 °C.

### 2.5. Determination of the Entrapment Efficiency

Two different procedures were used for the entrapment efficiency determination:

(1) SLN preparation (0.4 mL) was purified by size-exclusion liquid chromatography. Sephadex-LH20 (1.0 × 10 cm) was employed as a stationary phase, eluted in sequence with water (15 mL), water:EtOH (50:50; 10 mL), EtOH (10 mL) and acetone (10 mL). This latter eluate allowed for the recovery of unentrapped (free) BXL, while the aqueous eluate contained the entrapped BXL. The fraction eluted with water was treated with acetone in order to destroy the SLN and release the BXL [21].

(2) The SLN preparation (3 mL) was centrifuged at 105,000× *g* at 4 °C for 90 min using an Ultima TL Ultracentrifuge (Beckman, Milan, Italy). The amount of the free BXL present in the suspension was determined from the supernatant properly diluted with acetone (1:3). The entrapped BXL was determined from the pellet, employing acetone (2 mL) to destroy the nanoparticles.

Both trapped and free BXL recovered with the two above-described approaches (performed in triplicate) was detected by HPLC-UV (Agilent, Series 1100, Milan, Italy) equipped with a diode array detector set at 280 and 390 nm; a Luna C-18 (Phenomenex; 5 μm, 250 mm × 4.60 mm) was employed as column. The mobile phase consisted of a gradient of CH_3_CN (solvent A) in water (solvent B) with a flow rate of 1.0 mL/min: t_0_A = 10%, t_13_A = 100%, t_15_A = 100%, t_25_A = 10%. In these conditions, BXL eluted at about t_R_ = 11.99 min. The concentration of BXL was calculated by external standard calibration, comparing the area of the peak of BXL in selected samples with BXL standard solutions of known concentrations.

The entrapment efficiency % (EE%) was calculated indirectly and directly according to Equations (1) and (2), respectively [22]:

(1) $$\mathrm{EE}\%=[(\mathrm{mg}{\mathrm{BXL}}_{tot}-\mathrm{mg}{\mathrm{BXL}}_{free})÷\mathrm{mg}{\mathrm{BXL}}_{tot}]\times 100$$

(2) $$\mathrm{EE}\%=(\mathrm{mg}{\mathrm{BXL}}_{entrapped}÷\mathrm{mg}{\mathrm{BXL}}_{tot})\times 100$$

### 2.6. In Vitro Release Study of BXL from SLN

The in vitro release study of BXL was carried out for one week. Briefly, 1 mL of SLN formulation containing BXL equivalent to 0.4 mg/mL was placed into a dialysis tube with a molecular weight cut-off of 3.5 kDa (Spectra/Pro, Spectrum Lab., Rancho Dominguez, Ca, USA) and dispersed in a beaker containing 30 mL of H_2_O/EtOH 80:20 mixture. The solution was stirred at 200 rpm at 37 ± 0.5 °C. At pre-determined intervals, 1 mL of the release medium was withdrawn and replaced with an equal volume of fresh release medium. The concentration of BXL was determined by HPLC.

### 2.7. Antioxidant Activity Determination

#### 2.7.1. DPPH^•^ Scavenging Assay

The 2,2-diphenyl-1-picrylhydrazyl radical scavenging activity (DPPH^•^) of BXL and BXL-loaded SLN (SLN 8) samples was determined with the procedure previously reported [23]. A 150 mM DPPH^•^ solution (in MeOH) was freshly prepared and added (200 µL) to 96-well plate, then, aliquots (10, 20, and 30 μL) of samples (1.2 mM) were added to the 96-well plate. The mixtures were stored in the dark at 25 °C, and the OD was acquired with Synergy H1 microplate reader (Agilent, Milan, Italy) at 517 nm after 30 min. Quercetin was employed as reference compound, and all the experiments were performed in triplicate. Aliquots (10, 20, and 30 μL) of MeOH were added to DPPH^•^ solution and these samples were employed as negative control.

The percentage of DPPH^•^ quenched was calculated according to Equation (3):

(3) $$quenched\;{\mathrm{DPPH}}^{•}\;\%=\frac{\left(OD_{ctrl}-OD_{sample}\right)}{OD_{crtl}}\times 100$$

where *OD_ctrl_* and *OD_sample_* are the optical density of the DPPH^•^ solution without and in presence of the samples, respectively. EC_50_ is the effective concentration (μM) required to quench the 50% of DPPH^•^ present in solution, and it was calculated by regression analysis of quenched DPPH^•^ %.

#### 2.7.2. Oxygen Radical Absorption Capacity (ORAC) Assay

The ORAC assay was performed as previously reported [23]. All the regents used were freshly prepared. Trolox (used as the standard in the range 5–50 µM), quercetin (used as positive reference; 2.4 mM) or samples (diluted to gain several concentrations: 1.2, 0.6 and 0.1 mM) were added (25 µL) to a black walled 96-well plate. Then, fluorescein (8.16 × 10^−5^ mM in 75 mM phosphate buffer, pH = 7.4; 150 μL) was added to the multi-well plate and this was shaken at 37 °C for 10 min. The reaction started with the addition of a 153 mM 2,2′-azobis(2-methylpropionamidine) dihydrochloride solution (25 μL). Fluorescence intensity was measured every 1 min for 31 cycles at λEx = 485 nm, λEm = 528 nm, GAIN 50. Tris-HCl buffer solutions (10–80 µM) were used for the negative control. The data acquired in triplicate for each sample were elaborated as the area under the curve (AUC), and Trolox was used as the standard to obtain a calibration curve (R^2^ = 0.9971), in order to determine the ORAC value of the samples as Trolox equivalents (TE) with Equation (4):

(4) $$\mathrm{ORAC}=\frac{\left(AUC_{sample}-AUC_{blank}\right)}{\left(slope\;AUC_{Trolox}\right)}\times \frac{1}{\left(molarity\;sample\right)}$$

### 2.8. Stability Studies

To investigate the stability, SLN were stored in hermetically sealed bottle at room temperature for three months. Technological parameters (particle sizes, PDI and ZP values) were determined after 24 h, a week and at the end of the 1st, 2nd and 3rd months.

### 2.9. Preparation of MLV

MLV were prepared both in the absence and the presence of BXL at different molar fractions (0.003; 0.015; 0.03; 0.045; 0.06; 0.09 and 0.12). DMPC was dissolved in Chloroform/Methanol (1:1, *v*:*v*) while BXL was dissolved in Acetone. Aliquots of the DMPC solution containing 7 mg of DMPC were delivered into glass tubes in which aliquots of the BXL solution were added in order to have the exact molar fraction of the compound with respect to DMPC. The solvents were evaporated under nitrogen flow (in a water bath at 37 °C) to obtain the lipid films that were freeze dried overnight. The films were hydrated with 168 µL of 50 mM TRIS (hydroxymethyl)-aminomethanesolution (pH = 7.4), heated in a water bath at 37 °C for 1 min, vortexed for 1 min (the procedure was repeated three times) and kept at 37 °C for 1 h [24].

### 2.10. Differential Scanning Calorimetry

Calorimetric analysis was performed using a Mettler Toledo STAR^e^ thermoanalytical system (Greifensee, Switzerland) equipped with a DSC1 calorimetric cell. A Mettler TA-STAR^e^ software (version 16.00) was used to obtain and analyze data. The sensitivity was automatically chosen as the maximum possible by the calorimetric system. The calorimeter was calibrated using Indium (99.95%), based on the setting of the instrument. Aluminum calorimetric pans of 160 µL were used. Enthalpy changes were calculated from the peak areas.

#### 2.10.1. Unloaded and BXL-Loaded-SLN Analysis

The thermotropic behavior of the SLN was evaluated submitting the samples to DSC analysis under N_2_ flow (60 mL/min) as follows: a heating scan from 5 to 85 °C, at 2 °C/min; a cooling scan from 85 to 5 °C, at 4 °C/min; for at least three times to confirm the reproducibility of data.

#### 2.10.2. Unloaded and BXL-Loaded-MLV Analysis

Aliquots of 120 µL of MLV at different molar fractions of BXL (0.003; 0.015; 0.03; 0.045; 0.06; 0.09; 0.12) were situated in 160 µL DSC aluminum pans, which were hermetically sealed and subjected to calorimetric analysis under N2 flow (60 mL/min) as follows: a heating from 5 °C to 37 °C, at 2 °C/min; a cooling from 37 °C to 5 °C, at 4 °C/min.

The process was repeated three times to check the reproducibility of results. Unloaded MLV were also analyzed to be used as reference [25].

#### 2.10.3. MLV/SLN Interaction

An amount of 60 µL of MLV and 60 µL of SLN or BXL-loaded-SLN was placed in a 160µL DSC aluminum pan, which was hermetically sealed and subjected to calorimetric analysis under N2 flow (60 mL/min) as follows: (1) a heating scan between 5 and 85 °C at the rate of 2 °C/min; (2) a cooling scan between 85 and 37 °C at the rate of 4 °C/min; (3) an isothermal period of one hour at 37 °C and (4) a cooling scan between 37 and 5 °C (4 °C/min). This procedure was repeated eight times.

## 3. Results and Discussion

### 3.1. Formulation and Characterization of SLN

Natural products and their derivatives have played an important role in treating and preventing human diseases since ancient times and they still represent a significant source of modern drugs. However, their efficacy can be limited due to their low hydrophilicity and instability. In addition, they can show scarce absorption, poor pharmacokinetics and poor bioavailability. Novel nanoformulations based on drug-delivery systems could overcome these limitations. Nanoparticles have emerged as versatile, biodegradable, biocompatible carriers for the delivery of drugs [26,27]. From many studies, nanoformulations with sustained release and improved bioavailability at much lower doses than conventional preparations, and in many cases presenting a better safety profile, have been obtained. In this context, we designed SLN using PrecirolATO 5, Tegin-O and Oleth-20 for the encapsulation of BXL to overcome its biopharmaceutical limitations. The components were selected for their wide use, biocompatibility, biodegradability, and versatility [19,26] and on the basis of a series of preliminary experiments.

The formulated SLN showed appreciated consistency. As reported in Table 2, unloaded SLN, SLN 4 and SLN 8 showed a mean diameter of around 300 nm, and a polydispersity index within the acceptable range (<0.5) [28]. Zeta potential values were in the range −29 to −25 mV; the magnitude of zeta potential offers a signal of the stability of colloidal preparations. The surface charge of developed nanoparticles will allow their dispersion, since the range obtained is above the threshold values for agglomeration, i.e., ± 15 mV [29,30]. Thus, this surface charge is sufficient to confer physical stability to nanosuspension. The stability of BXL at the temperature used to prepare SLN has been confirmed by HPLC analysis.

The measurement of the sizes and the polydispersity index during the three months showed that the nanoparticle systems remained stable over the indicated period. The stability of the investigated SLN could be attributed to a steric stabilization due to the presence on the nanoparticle surface of long polyoxyethylene chains of oleth-20 used to prepare such SLN.

### 3.2. Entrapment Efficiency (EE)

The EE% of the SLN was determined by HPLC-UV, applying two different methodologies [19,20], thus allowing the quantification of both entrapped and free BXL after the preparation of SLN. Precisely, SLN preparation was loaded onto a Sephadex-LH20 column to afford an aqueous eluate containing the SLN and an acetone eluate containing the free BXL (method 1). The former eluate was diluted with acetone to destroy the SLN for the quantification of entrapped BXL. Alternatively, free BXL was determined after ultrafiltration from the permeate, whereas entrapped BXL in SLN was quantified from the retentate (method 2). The details are reported in the Experimental section. The entrapment efficiency (see Table 3) was calculated indirectly and directly according to Equations (1) and (2). The results achieved with both the methodologies agree with each other, thus confirming the reliability of the entrapment procedure. The results indicated an entrapment efficiency between 54.5 and 61.7% when SLN were loaded with 4 mg of BXL (SLN4) and between 62.8 and 68.7% when nanoparticles were loaded with twice the amount of BXL (SLN8).

### 3.3. In Vitro Release

**Figure 1:**
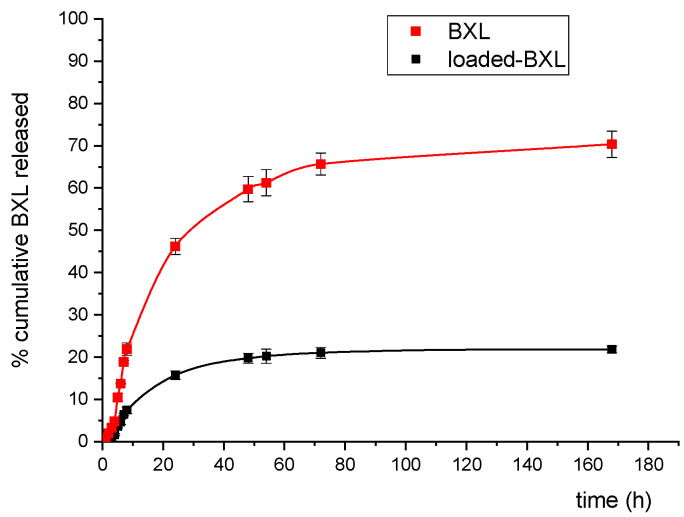
In vitro BXL release profile. In red is reported the quantification of BXL released from the formulation. In black is reported the quantification of BXL released based on the entrapment efficiency. The data plotted are means (*n* = 3) ± SD.

The in vitro release of BXL from SLN in H_2_O/EtOH 80:20 was evaluated by a dialysis method. An amount of 1 mL of medium was removed at predetermined time intervals and the released BXL was assayed by HPLC-UV [31]. The concentration of BXL released from the SLN was reported as a percentage respect to the total compound present in SLN and this value was plotted as a function of time in Figure 1. In figure, the BXL release taking into account the encapsulation efficiency (black line) and the BXL release from the SLN formulation (red line) are shown. The BXL is released faster within the 60 h followed by a slow release phase. Taking into account the encapsulation efficiency, the percentage of drug released reached 15.7% at 6 h and then it slowly increased up to 20.2% at 54 h, stabilizing at 21% for the remaining time (from 54 h up to 168 h). The percentage of drug released from the total SLN formulation reached the 60 % between 48 and 54 h and increased up to 70% within 168 h.

Similar values have been obtained for the polyphenolic quercetin [32,33]. The sustained release of BXL SLN could reveal the applicability of these SLN as a drug-delivery system which could permit the accumulation of the drug in the cells and allow the delivery of BXL for a long timeframe.

### 3.4. Antioxidant Activity Determination

Benzoxanthene lignans are reported for a variety of biological activities, including antioxidant activity [8]. In order to determine the capability of pure BXL and BXL-loaded in SLN to scavenge reactive oxygen species, two in vitro assays were employed. Usually, the most commonly employed methods to measure the antioxidant activity are split into assays based on hydrogen atom transfer (HAT) such as the ORAC assay, and others based on single electron transfer (SET) such as DPPH radical scavenging assay. The results of the two tests are reported in Table 4. DPPH radical scavenging activity is expressed as the concentration of sample quenching the 50% of radical (EC_50_), thus the lower the EC_50_ values the higher the activity. ORAC results are reported referring to the antioxidant standard Trolox (TE; µmol trolox equivalent), namely, higher TE value corresponds to higher antioxidant activity. Quercetin was employed as positive reference in both the assays. According to the results, BXL maintains its antioxidant activity even when it is loaded in SLN, and DPPH and ORAC values (Table 4) are similar to those of the free active compound. These results are in agreement with the findings on similar SLN formulations [34] and encourage future studies with BXL-SLN aimed at in vivo evaluation of antioxidant activity with a potential prolonging of the antioxidant effect.

### 3.5. Differential Scanning Calorimetry

DSC has an important role in the characterization of lipid nanoparticles and their interactions with biological membrane models. It is able to measure the heat exchanges associated with structural alterations of materials and allows us to obtain information about the structural properties of the samples [35]. In this study, DSC was used to characterize unloaded and loaded SLN and to study their interactions with liposomes of 1,2-dimyristoyl-sn-glycero-3-phosphocholine (DMPC). In addition, DSC was used to study the effect of BXL on the DMPC MLV.

#### 3.5.1. Unloaded and BXL-Loaded-SLN Analysis

**Figure 2:**
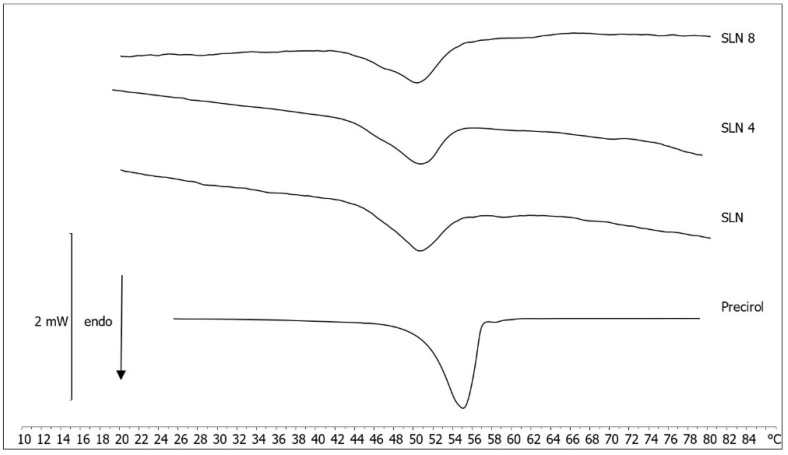
Calorimetric curves, in heating mode, of Precirol, SLN, SLN 4 and SLN 8.

Calorimetric curves of Precirol, unloaded SLN, SLN 4 and SLN 8 are shown in Figure 2. Precirol exhibits a peak at 55.06 °C and an enthalpy variation of 125 J/g. Unloaded SLN are characterized by a peak at around 51 °C and an enthalpy variation of −90.39 J/g. SLN melting temperature was about 4 °C lower than that of Precirol, which could be due to an increase in surface area resulting from SLN colloidal sizes and due to interactions between solid lipid and surfactant molecules that led to a less ordered structure. The enthalpy variation decrease and the ΔT_1/2_ increase are an indication that, during the melting, a lower cooperation among the lipid molecules occurs. The loading of BXL inside SLN did not produce significant variation of the melting temperature but it produced the appearance of a small shoulder at lower temperature; the shoulder was more accentuated when the concentration of BXL was higher. BXL also caused a slight decrease in the enthalpy variation and an increase in the ΔT_1/2_ (Table 5). The variation of the calorimetric curve of loaded SLN compared to unloaded SLN, as well as to the enthalpy variation and ΔT_1/2_, are an indication of the incorporation of BXL into the SLN structure. The presence of the shoulder suggests that BXL could be incorporated in the SLN not homogeneously. The SLN exhibit melting points (>50 °C) above body temperature, which is a prerequisite for retaining the solid state of the nanoparticles. In fact, many of the claimed advantages of SLN as drug carrier systems are related to the solid state of their lipid matrix [36].

#### 3.5.2. Unloaded and BXL-Loaded-MLV Analysis

**Figure 3:**
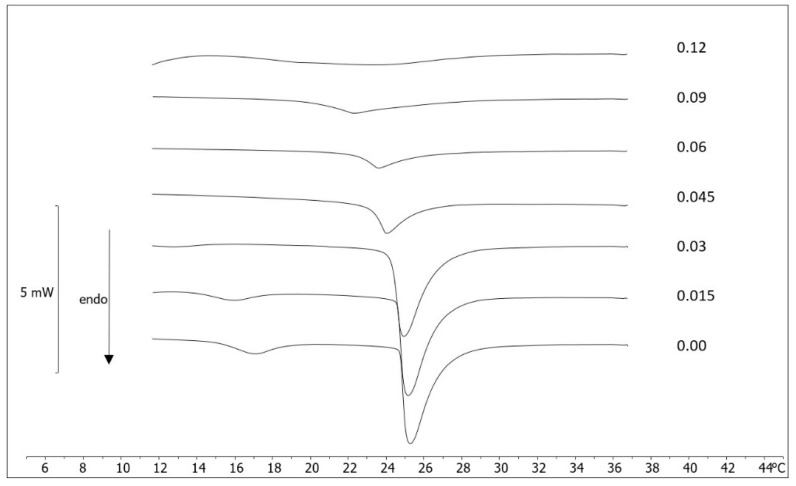
Calorimetric curves, in heating mode, of MLV prepared without and with BXL at different molar fractions.

Calorimetric curves of MLV without and with BXL at different molar fractions (0.00; 0.015; 0.03; 0.045; 0.06; 0.09; 0.12) are reported in Figure 3. The calorimetric curve of unloaded MLV is characterized by a pre-transition peak at about 17 °C (related to the transition from the lamellar gel phase to the ripple phase) and a transition peak at about 25 °C (related to the transition from the ripple phase to liquid crystalline phase) [37]. The increased concentration of BXL led to significant variation in the calorimetric curve of MLV; in fact, the pre-transition peak decreased and completely disappeared starting from the molar fraction of 0.03. The main transition peak shifted to a lower temperature and its intensity decreased. These data indicate that BXL affected the thermotropic behavior of MLV. It caused a fluidization of the MLV as well as a decrease in the cooperativity among the phospholipids during their melting. The presence of a unique peak indicates that BXL is homogenously incorporated in the DMPC MLV.

#### 3.5.3. MLV/SLN Interaction

**Figure 4:**
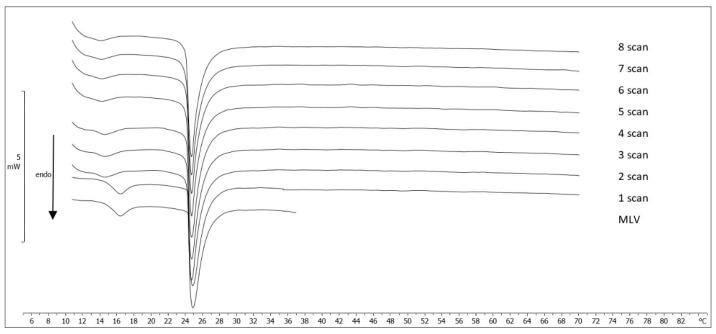
Calorimetric curves, in heating mode, of MLV put in contact with BXL at increasing incubation time. There are 68 min between one scan and the next.

This intricacy of the cell membrane structure makes the interactions with drugs and drug-delivery systems very difficult to investigate. Therefore, simplified artificial membrane systems, which mimic the natural bilayer lipid membrane, have been developed. In this article, DMPC MLV are used as a simplified system that mimics many biological membrane properties [38]. DSC was used to evaluate the interaction between SLN and MLV. Before evaluating this eventual interaction, we studied the ability of BXL to be incorporated into MLV when put in contact with them. For this reason, BXL and MLV were put in contact in a calorimetric pan and submitted to DSC analysis. The calorimetric curves are shown in Figure 4. If BXL was able to be incorporated into the MLV, a variation of the calorimetric curve should be seen. In the figure, it is evident that only the pre-transition peak slightly varies, whereas the main peak remains unchanged during all the contact time with BXL. This result suggests that BXL does not affect the thermotropic behavior of MLV and it is not able to cross the aqueous medium, reach the MLV surface and go inside the MLV structure, probably due to its low water solubility.

**Figure 5:**
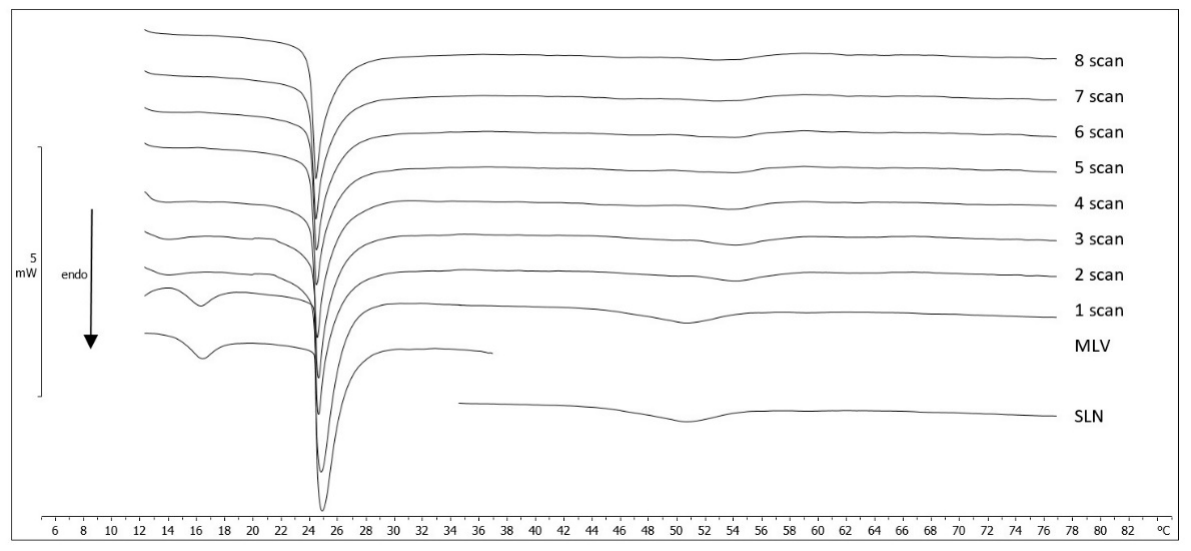
Calorimetric curves, in heating mode, of MLV put in contact with SLN, at increasing incubation time. There are 68 min between one scan and the next.

**Figure 6:**
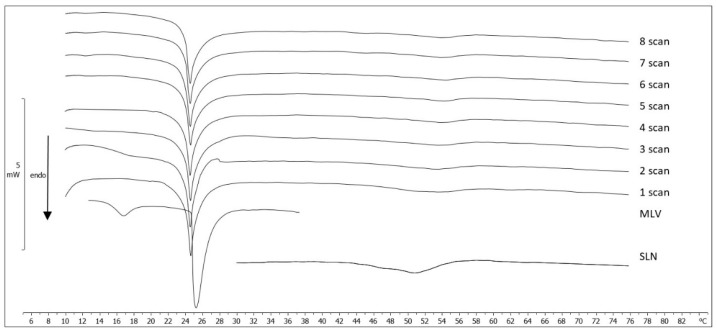
Calorimetric curves, in heating mode, of MLV put in contact with SLN 8 at increasing incubation time. There are 68 min between one scan and the next.

To evaluate the interaction between MLV and unloaded SLN or BXL-loaded SLN, the samples were put in contact in the calorimetric pan and submitted to analysis over a period of time. The interaction between MLV and SLN is highlighted by the variation of the calorimetric curves with respect to the calorimetric curve of the samples recorded before the contact. The calorimetric curves of the interaction between MLV and unloaded SLN show important features (Figure 5). First, we will consider the peaks of the MLV: the pre-transition peak decreases and then disappears; the main peak slightly shifts to a lower temperature and its intensity decreases. The peak of unloaded SLN shifts and the intensity decreases. These features are a sign of the interaction between MLV and SLN. In Figure 6, the calorimetric curves related to the interaction between MLV and SLN 8 are shown. As far as the MLV are concerned, the pre-transition peak vanishes from the first scan and the main peak slightly moves to a lower temperature and its intensity decreases. As the alteration of the phase behavior of MLV and SLN is due the presence of a perturbing agent in their structure, we can hypothesize that MLV and SLN 8 interact.

**Figure 7:**
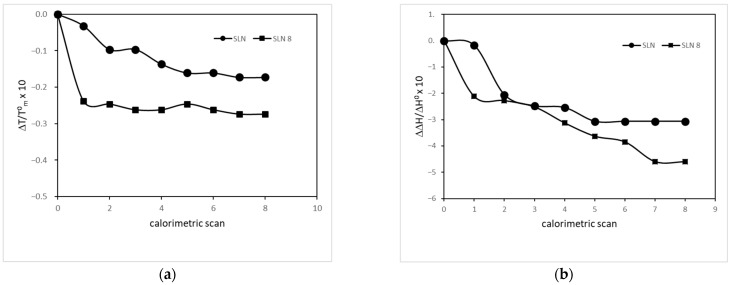
(**a**) Transition temperature variation of MLV left in contact with SLN and SLN 8, as a function of the calorimetric scans. There are a 68 min between one scan and the next. The transition temperature variation is reported as ΔΤ/T^0^_m_ (ΔΤ = T_m_ − T^0^_m_, where T_m_ is the transition temperature of the MLV left in contact with SLN or SLN 8 and T^0^_m_ is the transition temperature of MLV. (**b**) Transition enthalpy variation of MLV left in contact with SLN and SLN 8, as a function of the calorimetric scans. There are 68 min between one scan and the next. The transition enthalpy variation is reported as ΔΔH/ΔH^0^ (ΔΔH = ΔH − ΔH^0^, where ΔH is the transition enthalpy variation of the MLV left in contact with SLN or SLN 8 and ΔH^0^ is the transition enthalpy variation of MLV).

Let us compare the results obtained with SLN and SLN8. In Figure 7a, the transition temperature variation of MLV is reported as a function of calorimetric scans. A decrease in this parameter is evident and the decrease is more evident in the experiment run with SLN 8. The results related to the enthalpy variation as a function of the calorimetric scans, shown in Figure 7b, clearly indicate a decrease in the parameter and that the decrease is more pronounced in the experiment run with SLN 8. This information, together with the results of the MLV prepared with BXL, provide us with important information. We saw that BXL interacts with MLV. SLN and SLN 8 interact and affect the thermotropic behavior of MLV with SLN 8 exerting a more pronounced effect that can be due both to SLN and to BXL (SLN and BXL could exert an additive effect on MLV); in other words, SLN could enter the MLV and release BXL. The results of our research on the interactions between BXL-loaded SLN and a biological membrane model pointed out that SLN could be able to penetrate into the biomembrane, thus facilitating BXL permeation into the biomembrane itself. Therefore, SLN interactions with biomembranes could account for their ability to allow the penetration of BXL into cell membranes improving its uptake by the cells and, hence, its bioavailability.

## 4. Conclusions

In this work, a bioactive benzoxanthene was included in SLN, prepared by the Phase Inversion Temperature method, which produced promising results in terms of size, zeta potential, entrapment efficiency and stability. The percentage of benzoxanthene released from SLN was about 70%. The DPPH and ORAC tests indicated that BXL maintains its antioxidant activity even when it is loaded in SLN. Differential scanning calorimetry was used to study the SLN thermotropic behavior and interaction with biomembrane models represented by multilamellar vesicles of dimyristoylphosphatidylcholine. The obtained data suggest that SLN could interact and release the benzoxanthene into the biomembrane model. The results obtained in this study suggest the potential use of SLN as a delivery system for benzoxanthene.

## Figures and Tables

**Table 1:** Composition of unloaded and BXL-loaded SLN.

	SLN	SLN 4	SLN 8
BXL (mg)	-	4	8
PrecirolATO 5 (mg)	200	200	200
Tegin-O (mg)	126	126	126
Oleth-20 (mg)	246	246	246
H_2_O	up to 20 mL	up to 20 mL	up to 20 mL

**Table 2:** Dimension (Z-Ave), polydispersity index (PDI) and zeta potential (ZP) of unloaded SLN, SLN4 and SLN8.

	Z-Ave (nm ± SD)	PDI (-) ± SD	ZP (mV ± SD)
Unloaded SLN	305.4 ± 1.3	0.360 ± 0.04	−24.9 ± 0.1
SLN 4	296.1 ± 10.3	0.395 ±0.07	−25.6 ± 0.1
SLN 8	300.2 ± 6.9	0.368 ± 0.06	−28.9 ± 0.1

**Table 3:** Entrapment efficiency (EE) determination of BXL in SLN4 and SLN8. Data are reported as mean (*n* = 3) ± SD.

Sample	Method	EE% *
SLN 4	Sephadex-LH20	59.3 ± 2.0 ^a,b^ (Equation (1))
		61.7 ± 1.8 ^a,c^ (Equation (2))
	Centrifugation	54.5 ± 2.7 ^b,c^ (Equation (1))
		58.7 ± 0.9 ^a,d^ (Equation (2))
SLN 8	Sephadex-LH20	62.8 ± 4.1 ^b,d^ (Equation (1))
		66.1 ± 2.7 ^c,d^ (Equation (2))
	Centrifugation	65.5 ± 1.4 ^a,e^ (Equation (1))
		68.7 ± 3.9 ^b,e^ (Equation (2))

* Each determination was performed in triplicate. EE% was calculated with indirect method according to Equation (1) and with direct method according to Equation (2). ^a–e^ Different letters indicate statistical differences at *p* < 0.01 (Tukey test).

**Table 4:** Antioxidant activity determination of BXL and SLN8. EC_50_ values refer to DPPH radical scavenging activity; TE values refer to ORAC assay.

Sample	DPPH (EC_50_) ^1^	ORAC (TE) ^2^
BXL	68.7 ± 12.4 ^a,b^	6.2 ± 0.3 ^a,d^
SLN 8	83.1 ± 7.3 ^a,c^	4.4 ± 0.2 ^b,d^
Quercetin	17.9 ± 4.3 ^b,c^	15.4 ± 2.3 ^c,d^
MeOH ^3^	>350	-
Tris-HCl ^3^		0.05 ± 0.01 ^a,e^

^1^ Results are reported in μM as mean ± SD (*n* = 3). ^2^ Results are reported as Trolox equivalent (TE) in µmol as mean ± SD (*n* = 3). ^3^ Negative controls. ^a–e^ Different letters indicate statistical differences at *p* < 0.05 (Tukey test).

**Table 5:** Transition temperature (T_m_), enthalpy variation (ΔH) and peak width at half height (ΔT_1/2_) of Precirol, SLN, SLN4 and SLN8.

Sample	T_m_ (°C)	ΔH (J/gr Precirol)	ΔT_1/2_ (°C)
Precirol	55.06	−125.00	3.65
SLN	50.60	−90.39	5.79
SLN 4	50.63	−76.32	5.92
SLN 8	50.44	−73.31	6.13

## Data Availability

Data were generated at the Department of Drug and Health Sciences and at the Department of Chemical Sciences, University of Catania. Data supporting the results of this study are available from the corresponding authors on request.
